# Death Accelerated by Salivation in the Third Stage of Tubercular Consumption

**Published:** 1848-11

**Authors:** 


*Death Accelerated by Salivation in the Third Stage of Tubercular Consumption.*—An inquest was held on the 11th of August on the body of Mrs. James Perry, whose death was reported to have been accelerated by the improper exhibition of mercury, said to have been administered by an unlicensed practitioner named Young. The facts of the case, as elicited at the inquest, are simply these:—Mrs. P. had been, until a very recent period before her death, under the charge of Dr. Arnoldi, and Dr. Holmes had seen her in consultation. The existence of tubercles in her lungs had been clearly made out; and for some time before the intermission of Dr. Arnoldi's attendance, a palliative treatment had been most judiciously adopted. In consequence of the advice of friends, Young was called in, who stated that her lungs were not diseased at all; that her liver was tuberculated, as well as her kidneys, and that the uterus was somehow or other displaced, and that the cessation of the catamenia, a common symptom in phthisis, was due to that displacement. This latter he endeavored to rectify by some kind of manual interference, some traces of injury from which were visible at the *post-mortem;* and he persuaded the patient to submit to a system of salivation, which he assured her would cure her. In the course of three or four weeks, however, despite Young's predictions, the patient breathed her last, having been visited for two or three days by Dr. Burns, who found her suffering from salivation, with its concomitants. The inquest was held to determine the propriety of Young's practice, and how far it may have accelerated the decease. Drs. Nelson and Hall having been summoned on the part of the Crown, performed the *post-mortem* examination, and found the left lung the seat of extensive tubercular deposit, with anfractuous cavities, as well as the superior portion of the right lung, with very general pleuritic adhesions on the left side, and superior portion of the right. The liver slightly enlarged, with a tendency to granular degeneration ; the mesenteric glands in a tuberculated state ; but all the other abdominal viscera of normal appearance. The uterus perfectly healthy, and in natural position. The upper part of the vagina, immediately behind the os uteri, presented an ecchymosed appearance, which Dr. Nelson considered as the effect of injury. Although the question of salivation was well substantiated, still there was not evidence adequate to bring the exhibition of the mercury home to the prescriber, the books of the apothecary, which were sent for and examined, failing in the proof, in consequence of prescriptions having been compounded for several persons named Mrs. Perry by Young's orders. The verdict returned was "that the death of the deceased was accelerated by the improper administration of mercury, but there is no evidence to prove by whom such mercury was exhibited." This verdict was strictly in accordance with what was elicited at the inquisition, and we can therefore find no fault with it, because the jury could not travel beyond their record ; but we have no doubt, that had the evidence been conclusive, the verdict of the jury would have inculpated Mr. Young to rather an alarming extent. Young has escaped in the mean while, and *in the mean while* he prosecutes his trade, not, most certainly, to the damage of any practitioner in this city, but most assuredly to the jeopardy of *some* who have since placed themselves in his hands.—*British American Journal of Medical and Physical Science.*

